# HIV-specific Th2 and Th17 responses predict HIV vaccine protection efficacy

**DOI:** 10.1038/srep28129

**Published:** 2016-06-21

**Authors:** Delphine Sauce, Guy Gorochov, Martin Larsen

**Affiliations:** 1 Sorbonne Universités, UPMC Univ Paris 06, INSERM, Centre d’Immunologie et des Maladies Infectieuses (CIMI-Paris UMRS 1135), F75013, Paris, France; 2 AP-HP, Groupement Hospitalier Pitié-Salpêtrière, Département d’Immunologie, F75013, Paris, France

## Abstract

Understanding the factors that delineate the efficacy of T-cell responses towards pathogens is crucial for our ability to develop potent therapies and vaccines against infectious diseases, such as HIV. Here we show that a recently developed analytical tool, the polyfunctionality index (PI), not only enables prediction of protection after vaccination against HIV, but also allows identification of the immunological pathways involved. Our data suggest that induction of a synergistic network of CD4^+^ T-cell subsets is implicated in HIV-protection. Accordingly, we provide evidence that vaccine-induced protection is associated with CD40L expressing Th2 cells and IL-2 secreting Th17 cells. In conclusion, we describe a novel approach that is widely applicable and readily interpretable in a biological and clinical context. This approach could greatly impact our fundamental understanding of T-cell immunity as well as the search for effective vaccines.

Efficacious vaccines against major pathogens, such as HIV, are presently lacking. However, comprehensive quantitative and qualitative immunological data of individuals showing partial response to vaccine candidates are available, such as T-cell polyfunctionality measures^1^,^2^,^3^. T-cell polyfunctionality identifies T-cell subsets capable of simultaneously producing defined combinations of effector molecules, such as cytokines and chemokines. Initial analysis of the preventive HIV-vaccine trial RV144^4^ revealed no individual functional CD4^+^ T-cell correlates of vaccine protection (IFN-γ, IL-4, IL-17A, IL-2, CD40L and TNF-α)^2^. However, a recent study demonstrated that prediction models based on polyfunctionality scores derived from a Bayesian hierarchical framework model, identified subjects protected from subsequent HIV-contraction^3^. Of note, it was not determined, which effector molecules were responsible for the predictive capacity.

We previousy developed a polyfunctionality index (PI), which can desiccate the predictive capacity of individual and combined effector molecules^5^. PI-analysis relies on an algorithm, which scores T-cell subsets according to their level of polyfunctionality, based on exhaustive boolean combination analysis of measured effector molecules, such as cytokines. We and others have previously shown that simultaneous expression of certain effector molecules has a positive impact on T-cell efficacy *in vitro*^6^ and *in vivo*^1^. The polyfunctionality index was constructed so that such synergistic mechanisms may be modelled, thereby allowing a more appropriate description of host/pathogen interaction^7^,^8^. Indeed, the PI framework includes immunologically interpretable parameters, which can be adjusted in order to improve PI-based prediction models (parameter adjustment is described in the materials and method section). We recently demonstrated that PI analysis^5^ of T-cell populations, allows not only to correlate combinatorial effector functions with biological outcomes, but also to appreciate the differential impact of individual effector functions on T-cell efficacy^8^. This was accomplished by regressing measures of T-cell efficacy, such as T-cell target killing and pathogen control, on empirical proportions of polyfunctional T-cell subsets (T-cell quality metric), analyzed within the framework of the PI in a “supervised” manner.

Here we show that PI-analysis not only enables prediction of protection after vaccination against HIV, but also allows clinically and immunologically meaningful interpretations. Our data highlight that induction of a synergistic network of HIV-specific CD4^+^ T-cell subsets, composed of CD40L-expressing Th2 cells and IL-2-secreting Th17 cells, is associated with HIV-protection.

## Results and Discussion

Beyond being the first HIV vaccine trial showing some protective effect against HIV contraction, the RV144 study is also one of the largest and most thoroughly analyzed. Indeed, 16402 HIV-seronegative volunteers with high risk of HIV-infection were randomized to vaccination or placebo treatment. Functional analysis of HIV-specific CD4^+^ T-cells was subsequently conducted by *in vitro* HIV-antigen stimulation, cell staining and flow cytometric detection of a comprehensive panel of CD4^+^ T-cell effector functions (IFN-γ, IL-4, IL-17A, TNF-α, IL-2 and CD40L) at the single-cell level^2^.

CD4^+^ T-cells can be categorized in T helper (Th) cell subsets according to the expression of a set of transcription factors, including T-bet (Th1), GATA3 (Th2) and ROR-γt (Th17)^9^. These transcriptional profiles translate into functional profiles, which are dominated by certain central cytokines such as IFN-γ (Th1), IL-4 (Th2) or IL-17A (Th17)^10^, herein termed prototypic effector molecules. We have previously described how non-supervised hierarchical cluster analysis of raw single-cell flow cytometry data can segregate CD4^+^ T-cells into their corresponding Th-type cell subsets^11^. Th-type cell subsets play highly divergent roles in host immunity ranging from pro-inflammatory to regulatory activity^12^. The PI is designed in such a way that parameters (*q* and *φ*) regulate to which degree effector molecules are considered to have additional or even synergistic effect. Briefly, the impact of polyfunctionality and individual effector molecules are assessed by *q* and *φ*, respectively. This design is well suited to analyze cells expressing effector molecules that work in concert to attain a particular biological effect. Conversely, the design is not conceived to represent systems where different cell types undertake opposing tasks. Therefore, to fully exploit the polyfunctionality index analysis, prototypic markers should be first employed to segregate CD4^+^ T-cells into Th1, Th2 and Th17 cell subsets, which would then be subjected individually to PI analysis of CD40L, IL-2 and TNF-α expression within these Th subsets. Here, we asked whether PI analysis of isolated Th subsets would give more appropriate inference on their respective roles in protecting against HIV-contraction.

**Figure 1:**
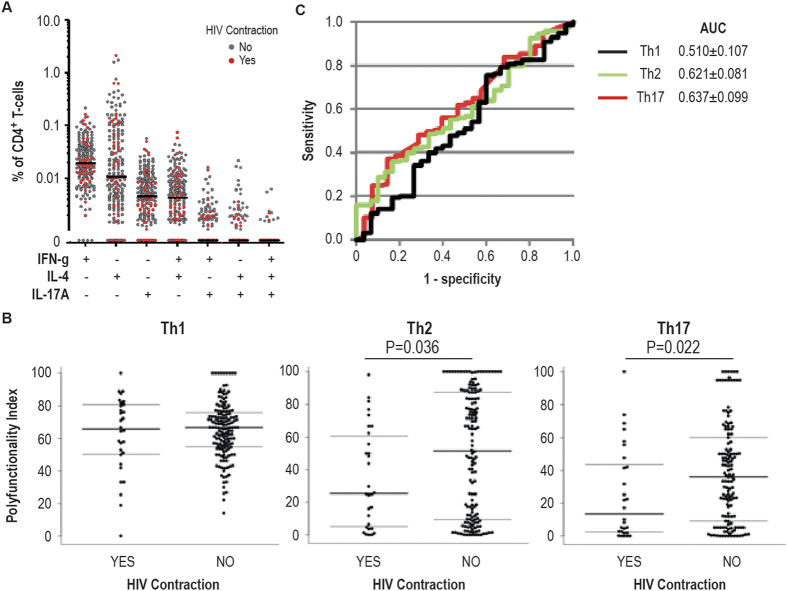
Th2 and Th17 cells are associated with HIV protection. (**A**) Frequency analysis of the 8 cell subsets comprised of the combination of 3 prototypic effector molecules, IFN-γ, IL-4 and IL-17A expressed by CD4^+^ T-cells stimulated with HIV antigens. Samples were stratified according to HIV contraction in the 42 months follow-up period post vaccination. The median of all samples are marked with a black line. (**B**) The functional parameters, CD40L, IL-2 and TNF-α, were analyzed within stimulated CD4^+^ T-cells expressing IFN-γ (Th1), IL-4 (Th2) and IL-17A (Th17), respectively. Binary logistic regression models of the dependent variable HIV protection versus 3 independent variables, *qφ*-adjusted PI for Th1 (black, *q* = 0, *φ*_*CD40L*_ = 0, *φ*_*IL2*_ = 1000, *φ*_*TNFa*_ = 0), Th2 (green, *q* = 0, *φ*_*CD40L*_ = 1000, *φ*_*IL2*_ = 0, *φ*_*TNFa*_ = 0) and Th17 (red, *q* = 0, *φ*_*CD40L*_ = 20, *φ*_*IL2*_ = 375, *φ*_*TNFa*_ = 0) cell subsets were established. Receiver operating characteristic (ROC) curves for *qφ*-adjusted PI for each Th-subset are displayed. Prediction models based on the polyfunctionality of Th1, Th2 and Th17 cell subsets included 185, 180 and 165 of 226 vaccinated volunteers with detectable Th subset HIV-specific CD4^+^ T-cell responses, respectively. (**C**) The *qφ*-adjusted PI of Th1, Th2 and Th17 cell subsets were compared between volunteers stratified according to HIV contraction. Group comparisons were conducted with a non-parametric Mann-Whitney test. The area under curve (AUC ± 90% Confidence Interval) metric of each prediction model is indicated.

The PI was therefore separately calculated for Th1, Th2 and Th17 cell populations discriminated by their respective prototypic markers, IFN-γ, IL-4 and IL-17A. Of note, plastic cells producing more than one prototypic cytokine are included repetitively in multiple Th-subsets (Fig. 1A) to take into account their divergent functional potential. We included 185, 180 and 165 individuals with detectable Th1, Th2 and Th17 HIV-responses, respectively (cf. Table 1). To identify the importance of individual effector molecules and their synergy for HIV-vaccine efficacy we adjusted PI parameters to obtain the best predictive model of HIV protection for each Th-subset. The adjusted parameters *q* and *φ* reflect the predictive importance of polyfunctionality and individual effector molecules, respectively (cf. materials and methods). Using the *qφ*-adjusted PI (Th1, *q* = 0, *φ*_*CD40L*_ = 0, *φ*_*IL2*_ = 1000, *φ*_*TNFa*_ = 0; Th2, *q* = 0, *φ*_*CD40L*_ = 1000, *φ*_*IL2*_ = 0, *φ*_*TNFa*_ = 0 and Th17, *q* = 0, *φ*_*CD40L*_ = 20, *φ*_*IL2*_ = 375, *φ*_*TNFa*_ = 0) we observed higher polyfunctionality index levels of Th2 and Th17 cells within non-infected versus infected vaccinees (*P* = 0.036 and *P* = 0.022, respectively), suggesting a protective effect of functional Th2 and Th17 cells (Fig. 1B and Supplementary Fig. 1A). Accordingly, Th2 (AUC = 0.621) and Th17 (AUC = 0.637) also tended to provide better PI prediction of protection against HIV contraction than Th1 cells (AUC = 0.510, *P* = 0.18 and 0.15, respectively, Fig. 1C). Also, a prediction model including *qφ*-adjusted PI of both Th2 and Th17 cells did not significantly improve the prediction models (AUC = 0.672). Of note, when added to the models, anti-HIV envelope IgA titer, gender and baseline behavioral risk score showed no significant confounding effect on the prediction models. Importantly, the *qφ*-adjustment also revealed that the predictive capacities of Th2 and Th17 cells were tightly linked with CD40L and IL-2 expression, respectively, as determined by their respective *φ*-values. Indeed, restricted to the three effector functions, CD40L, IL-2 and TNF-a, Th2 and Th17 cells seem to predict HIV protection based on one dominant effector function (CD40L and IL-2, respectively) without any detectable impact of polyfunctionality (*q* = 0). To confirm this finding we then compared the frequency of CD40L, IL-2 and TNF-α expression within each Th-subset (Supplementary Fig. 1B), demonstrating that vaccinees contracting HIV have lower CD40L expression within the Th2 cell subset (*P* = 0.037) and lower IL-2 expression within the Th17 cell subset (*P* = 0.033), compared to those not contracting HIV (Supplementary Fig. 1B). Indeed, the frequency of CD40L expression within the Th2 cell subset (AUC = 0.621) and of IL-2 expression within the Th17 cell subset (AUC = 0.627) predicted protection against HIV contraction (Supplementary Fig. 1C). Our results suggest that CD40L-expressing Th2 cells, which are crucial for anti-HIV neutralizing antibody production^13^,^14^, and IL-2-expressing Th17 cells, which are instrumental in mucosal tissue responses^15^, may importantly contribute to protection against HIV infection. Of note, we did not find correlation between IgG and IgA antibody titers to HIV envelope antigen and the frequency of CD40L-expressing Th2 cells; however we do not have access to measures of antibody affinity nor the neutralizing capacity of the measured antibody responses.

It should be stressed that while vaccinated volunteers contracting HIV can be defined as non-protected, non-contraction of HIV could be the result of either protection or non-exposure to HIV. With this in mind, it is interesting to note that within the non-infected group of vaccinees, the frequencies of CD40L-expressing Th2 cells and IL-2-expressing Th17 cells follow a bimodal distribution (Supplementary Fig. 1B). This observation raises the possibility that the “truly protected” individuals would be those with relatively abundant CD40L^+^ Th2 and IL-2^+^ Th17 cell subsets. It would, therefore, be interesting to stratify patients according to these parameters and to re-evaluate HIV contraction at a later time point. These results underscore the protective effect of a compartmentalized polyfunctional CD4^+^ T-cell network and the importance of vaccine polyvalency.

In conclusion, we believe that PI is an effective method to determine T-cell quality derived predictive correlates of vaccine protection. PI is broadly applicable and intuitive to apply and interpret, while its resulting parameters, when adjusted to optimize prediction model fit, can provide direct biological insight. In the presented study the PI analytical approach successfully dissected the impact of polyfunctional T-cells and identified an important functional lymphocyte compartmentalization predictive of HIV protection. Our analysis suggests that data mining of previous vaccine trials may expose important predictive immune correlates of protective immunity, providing biomarkers and biological insights, which may guide the development of future HIV vaccines and ultimately an HIV cure.

## Material and Methods

Data for this study derive from a clinical trial protocol, which was approved by the relevant institutional review boards (The involved institutions are indicated here: https://www.clinicaltrials.gov/ with registration number NCT00223080). All study participants provided written informed consent for immune response exploratory analyses, and our study was carried out in accordance with relevant guidelines and regulations.

### HIV vaccine protection (RV144 vaccination trial)

The dataset from the RV144 trial was obtained from the online repositories provided in Lin *et al*^3^. Here we analyzed 226 volunteers enrolled in the RV144 trial. Volunteers were injected with a combination of ALVAC HIV (vCP1521) and AIDSVAX B/E (only weeks 12 and 24) at weeks 0, 4, 12, and 24, and immune responses at week 26 were evaluated as immune correlates of infection risk. Briefly, systemic IgA and IgG antibody titers to HIV envelope antigen were determined and CD4^+^ T-cells were stimulated or not with a peptide pool covering the HIV envelope protein contained in the vaccine and analyzed for their expression of IFN-γ, IL-4, IL-17A, TNF-α, IL-2 and CD40L at the single-cell level by flow cytometry^2^. All volunteers were followed for 42 months following the last vaccination and analyzed for HIV contraction^4^. In total, 38 of the 226 vaccinated volunteers contracted HIV.

### Polyfunctionality analysis

The RV144 datasets included exhaustive boolean combination gating of all functional parameters measured at the single-cell level on stimulated and non-stimulated CD4^+^ T-cells. Datamining of the 2^n^ (n being the number of bimodal parameters) boolean combination gates of a given dataset was conducted with the Boolean Datamining component of the Funky Cells ToolBox software (Version 0.1.2, www.FunkyCells.com). This was particularly important for segregation of Th1, Th2 and Th17 cells within the RV144 dataset based on the expression of prototypic effector molecules IFN-γ, IL-4 and IL-17a, respectively. Samples with undetectable HIV-specific CD4^+^ T-cell responses were identified as those where the frequency of cells expressing at least one of the measured effector molecules post HIV-antigen stimulation was below or equal to the same sample without stimulation. For the Th1, Th2 and Th17 cell subset analysis we finally excluded samples for which the corresponding prototypic effector molecule was below background level (cf. Table 1). The Th sub-analysis was conducted on the three effector molecules, CD40L, IL-2 and TNF-α. Scatter plot graphics were accomplished with the “beeswarm” package for R.

### Polyfunctionality Index analysis

The general polyfunctionality index (1) was previously described^5^,^8^. Briefly, the general polyfunctionality index was invented to reduce a 2^n^-dimensional polyfunctional measure to a one-dimensional value taking into account both individual and combined expressions of effector molecules. The index comprises a weighed sum of the frequencies of all 2^n^ (poly)functional T-cell subsets. Two weights are assigned to each individual functional T-cell subset. One weight increases with the number of simultaneously expressed effector molecules. This weight includes the parameter *q*, which when increased modulates the weight to enhance discrimination between less and more polyfunctional T-cell subsets. The second weight is based on the parameters, *φ*_*i*_, which assign differential weights to individual T-cell subsets depending on the expression of given effector molecules. Indeed, each effector molecule is associated with a unique *φ*. We had the following requirements for the general polyfunctionality index: A) It should range from 0 to 100 and B) It should accommodate different weights for all n functions as well as their combinations.


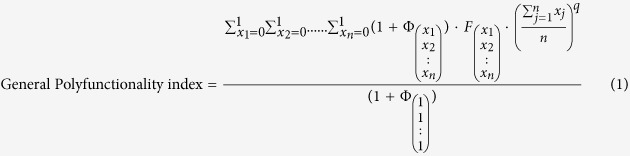



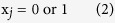



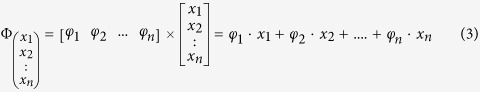



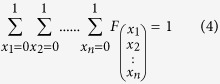



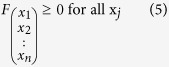


and


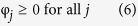


*n* > 0 is the number of functions studied. x_*j*_ indicates in a binary fashion (2) if the combinatorial T-cell subset 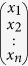 perform the j^th^ function (x_j_ = 1) or not (x_j_ = 0). 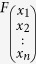 is the frequency of cells performing the particular combination of functions 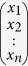. 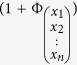 is a factor assigned to a T-cell subset performing the particular combination of functions 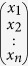 (3). *q* is the parameter that modulates the effect of increasing levels of polyfunctionality according to the number of simultaneous functions expressed by a given cell subset. The algorithm requires that the sum of all 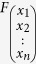 equals 1 (4) and that all 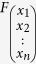 and all factors (*q* and *φ*_*j*_) are absolute values (5–6). High *q*-values will favour more polyfunctional T-cell subsets and high *φ*_*j*_-values will favour cell subsets positive for the j^th^ function, as previously described^5^,^8^.

### Statistical analysis

The general PI was used as an independent variable predicting the clinical end-point of interest (here HIV-contraction status) in a logistic regression model. Adjustment of the parameters *q* and *φ* to optimize the prediction model was based on an iterative approach. For combined *qφ*-adjustment a range of *q*- and *φ*-values at increments of 0.1 and 1, respectively, were imputed into the series expansions of the general PI. Maximum likelihood values were obtained for each *q* and *φ* combination resulting in a multidimensional matrix from which the optimal model fit was identified as the global maximum. We iterated *q* until 50 and *φ* until 1000. If there was an overly strong relationship between the end point and high *q* and/or *φ*-values, a local maximum might not have been reached. Indeed, when *φ* was found to be 1000 we tested that no further model improvement could be obtained by increasing *φ* further (10^3^–10^9^), while keeping other parameters fixed. Of note, adjusted *q*- and *φ*-values could subsequently be interpreted to identify which factors play essential roles for predicting vaccine efficacy as previously described^8^. Briefly, the *q*-value indicates the importance of polyfunctionality (*q* equal to zero indicates that there is no synergistic effect of polyfunctionality) and the *φ*-values represent the relative importance of individual effector molecules.

To visualize the predictive power of our logistic regression models we plotted receiving operator characteristic (ROC) curves, which depicts the specificity and sensitivity of each predicted value of a given model, Predictive accuracy was estimated as the area under the ROC curve (AUC). An AUC estimate approaching 1 is the result of a prediction model with maximal accuracy, whereas an AUC equal to 0.5 represents a predictive model with no predictive capacity. AUC from different prediction models were compared using the method by DeLong *et al*^16^. applied to both paired and unpaired sample sets^17^. All statistical analysis was performed with R (v3.1.3) and the pROC R package (v1.8)^17^. *P*-values less than 0.05 were considered statistically significant.

## Additional Information

**How to cite this article**: Sauce, D. *et al*. HIV-specific Th2 and Th17 responses predict HIV vaccine protection efficacy. *Sci. Rep.* **6**, 28129; doi: 10.1038/srep28129 (2016).

## Supplementary Material

Supplementary Information

## Figures and Tables

**Table 1:** Meta-analysis of 226 vaccinated volunteers.

Subset	Infected	Non-infected	Exclusion
Th1	30	155	41
Th2	30	150	46
Th17	28	137	61

Distribution of infection events and exclusion of volunteers with undetectable HIV-specific vaccine response.
