# Supplementary material for: HIV-specific Th2 and Th17 responses predict HIV vaccine protection efficacy
Source: Sci Rep. 2016 Jun 21;6:28129. doi: 10.1038/srep28129 (PMC4914845; doi:10.1038/srep28129)
Supplement: Supplementary Information [file srep28129-s1.pdf]

Supplementary information

HIV-specific Th2 and Th17 responses predict HIV vaccine protection efficacy.

Delphine Sauce[1], Guy Gorochov[1,2] and Martin Larsen[1,2]

[1]Sorbonne Universités, UPMC Univ Paris 06, INSERM, Centre d'Immunologie et des Maladies Infectieuses (CIMI-Paris UMRS 1135), F75013, Paris, France.
[2]AP-HP, Groupement Hospitalier Pitié-Salpêtrière, Département d'Immunologie, F75013, Paris, France.

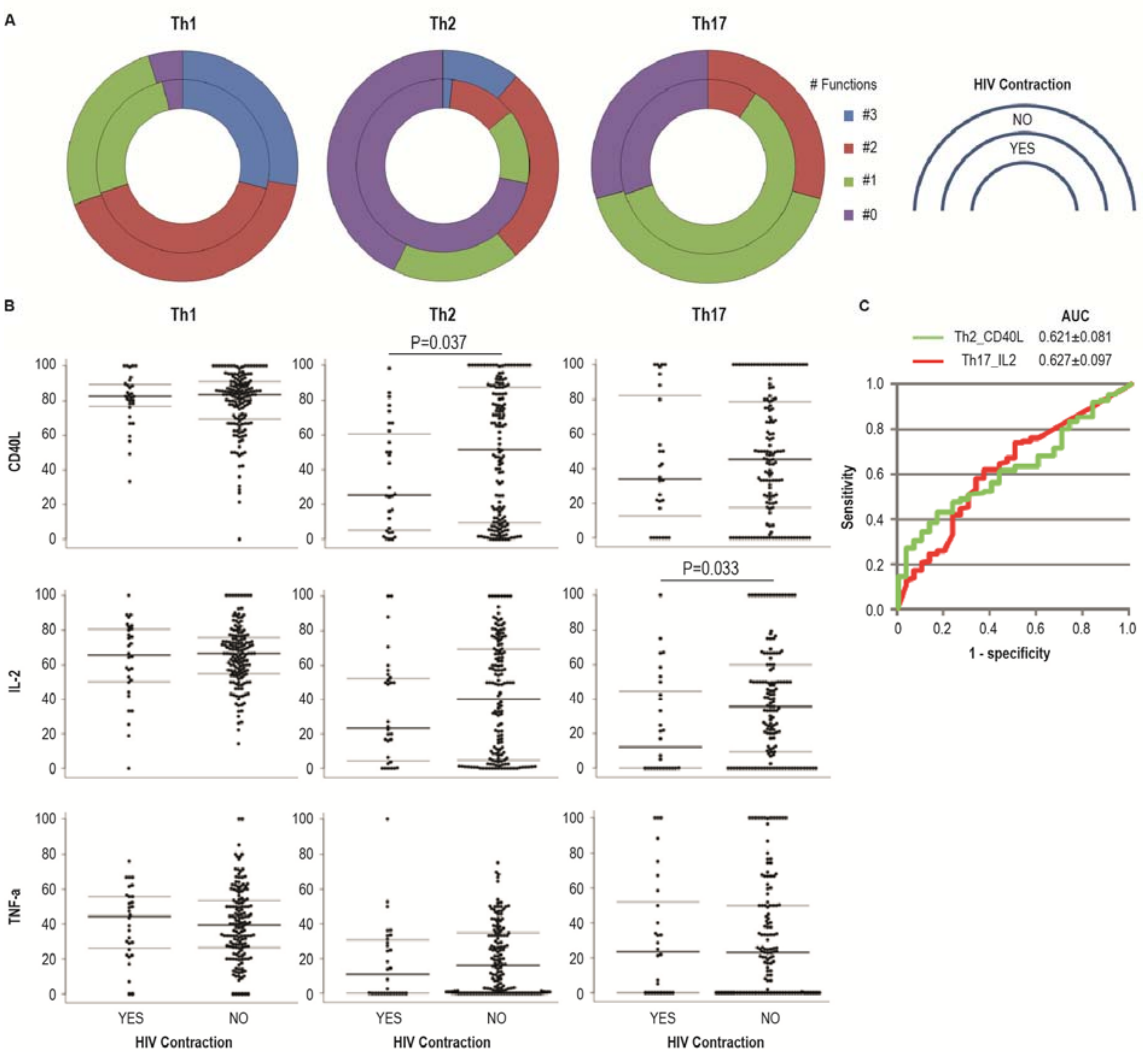

Supplementary Figure 1. Polyfunctionality of Th1, Th2 and Th17 cell subsets associated with HIV protection. A. Median polyfunctionality profile charts depicting the frequency of HIV-antigen stimulated Th1, Th2 and Th17 cells producing 1, 2 or 3 simultaneous effector molecules (CD40L, IL-2 and/or TNF-α) stratified according to HIV contraction status. **B.** Comparison of the percentage of Th1, Th2 and Th17 cells expressing CD40L, IL-2 and TNF-α stratified according to HIV protection status. **C.** ROC curves for HIV contraction predicted by the fraction of CD40L expressing Th2 cells (Th2_CD40L, green) and the fraction of IL-2 expressing Th17 cells (Th17_IL2, red). Group comparisons were conducted with a non-parametric Mann-Whitney test. The area under curve (AUC±90% Confidence Interval) metric of each prediction model is indicated.
